# The 5Cs of Positive Youth Development and Risk Behaviors in a Sample of Spanish Emerging Adults: A Partial Mediation Analysis of Gender Differences

**DOI:** 10.3390/ejihpe13110170

**Published:** 2023-11-01

**Authors:** Diego Gomez-Baya, Antonio David Martin-Barrado, Maria Muñoz-Parralo, Myunghoon Roh, Francisco Jose Garcia-Moro, Ramon Mendoza-Berjano

**Affiliations:** 1 Department of Social, Developmental and Educational Psychology, Universidad de Huelva, 21007 Huelva, Spain; antoniodavid.martin@dpee.uhu.es (A.D.M.-B.); maria.munoz988@alu.uhu.es (M.M.-P.); fjose.garcia@dpsi.uhu.es (F.J.G.-M.); ramon@uhu.es (R.M.-B.); 2 Department of Criminal Justice and Criminology, Salve Regina University, Newport, RI 02840, USA; myunghoon.roh@salve.edu

**Keywords:** youth, PYD, gender, cross-sectional, risk behaviors, Spain

## Abstract

Positive Youth Development (PYD) emerged as a holistic and strength-based perspective that focuses on the fact that young people may have the internal and external resources for healthy and successful development through five dimensions (5Cs) that empower them: Perceived Competence, Confidence, Character, Connection, and Caring. The aim of this study was to examine the relationship between the overall PYD factor, the 5Cs, and risk behaviors, in addition to analyzing gender differences. This study showed the results of a cross-sectional study of 1044 emerging adults from 11 Spanish universities in 2021. Data collection was performed by applying an online self-report measure. The results showed that the Character was protective against substance abuse, mainly in women, while the connection was related to the participation of betting money and online betting in men. Caring was protective against money bets in the men’s sample. However, controversial results were found regarding Perceived competence, which had a positive association with substance abuse, money bets, and drunk driving. It seems that high levels of Perceived competence, rather than objective competence, were associated with engagement in various risk behaviors. Concerning gender differences, men showed more risky behaviors than women. A partial mediation model pointed out that lower character and higher perceived competence in men partly explained the higher presence of risky behavior compared to women. These results underline the need to promote PYD within the university context to prevent risky behaviors by addressing gender differences and the separate role of the 5Cs.

## 1. Introduction

Each culture and sociohistorical moment explain the way in which young people become adults. In recent years, the delay in parenthood, as well as the longer time in education, has led to the existence of a very diverse new stage called emerging adulthood [1,2]. People in this stage of life have priorities and values that are different from those they had in childhood and adolescence, as well as a new search for identity [3]. Emerging adulthood can become a key stage that defines a person’s future and may present social, personal, school, and/or work challenges. In addition, unhealthy lifestyles and risky behaviors may emerge in adolescence and be consolidated during youth, which compromises personal well-being, health, and academic development, among other aspects [4,5,6].

In addition, the negative vision of adolescence prevailed in many social spheres in the 20th century, perhaps due to the “storm and stress” model introduced by Stanley Hall. In this line, deficit models emerged, which conceived of adolescence as a stage of problems, focusing on the weaknesses of young people. But evidence to date has underlined that this is not enough and that another more positive vision should be developed, paying attention to strengths rather than deficits [7,8,9,10,11]. Thus, the main challenge is to develop individual and community strengths in a context of social support with a progressive transfer of adult roles [6,11,12].

### 1.1. Positive Youth Development

In recent decades, a model emerged in the United States from positive psychology focused on competence development in youth contexts [13,14,15,16]. This new approach is known as Positive Youth Development (PYD), which highlights the developmental strengths that every person possesses [6,9,10,11,15,17]. It seeks to promote behaviors, skills, and competencies for successful academic, interpersonal, and occupational development [12,14,18]. The PYD model is complementary to the deficit model; the promotion of developmental opportunities promotes greater competence and indirectly steers the adolescent away from risky behaviors [14]. The assumption of a strength-based model has several advantages. First, it employs a new and positive vocabulary, with words such as psychological well-being, flourishing, and positive youth development [19,20]. This model encourages a new approach, giving young people an active role rather than considering them just passive recipients [21,22]. Some evidence for the PYD model has been collected In many countries, such as Spain.

One of the main and most comprehensive models based on this perspective is Lerner’s 5C model [17]. These are five interconnected components: Perceived competence, Confidence, Character, Connection, and Caring [23]. Perceived competence refers to the positive perception of self-efficacy in several areas. Confidence is closely related to self-esteem and a positive self-concept. Character is related to respect for social norms and culture. Connection refers to having positive relationships with others as well as being positively valued by others. Finally, Caring includes empathy and sympathy for others. If these components are fulfilled, contribution emerges, which is intimately related to a strong commitment to family, community, and environment and to own development [16,17,18,24].

### 1.2. Positive Youth Development and Risk Behaviors

As shown in the literature on this topic, successful development of the 5Cs is related to greater satisfaction with life, greater social commitment, and a lower presence of risky behaviors, which in turn encourages a healthier lifestyle [6,13,15,25,26]. Inversely, low fulfillment of the 5Cs is associated with greater problem behaviors such as sexual risk behavior, substance abuse, and antisocial behaviors [19]. Furthermore, there are some gender differences in relation to the PYD components, with males reporting more Perceived competence and Confidence (in line with male stereotypes, focusing on achievement and increased possibilities and experiences), while females report higher levels of Caring and Character (maybe due to gendered parenting styles characterized by greater emotional expressiveness and attachment) [25,27]. These differences were pointed out in Spanish youth samples by Manrique-Millones et al. [28] and Gomez-Baya et al. [29].

The abuse of alcohol, tobacco, and other drugs by young people can lead to diverse health problems and future chronic diseases, making this a public health problem. Because useful interventions are needed to face these problems, PYD stands out as one of the most efficient models, providing young people with opportunities for positive relationships with adults, constructive activities, and positive expectations. This promotion strategy allows for the development of self-regulation strategies and a greater sense of self-efficacy, which would increase the Cs and reduce risk factors or their adverse consequences [15,30]. Some authors highlighted the possible protective role of PYD against risky behaviors but concluded that more research is needed on the components involved [13,15]. Ceja et al. [31] stated that the key issue is to find out how to achieve the best development for each person while attending to their needs and capabilities. Furthermore, other authors indicated that the PYD model can be useful to reduce substance abuse and violent behavior, mainly in socially excluded youth with low educational levels, little social support, and few future aspirations. In this sense, it is important to highlight the protective role of social and emotional competence [32]. Moreover, it has been argued that each separate C of PYD can protect against different risky behaviors. However, a higher level of overall PYD was found to be associated with greater social integration, which may be marked by the consumption of alcohol and other substances that could be well socially accepted [33].

Finally, the PYD framework aims to educate and increase engagement and develop some indicators of thriving at the academic, psychological, and health levels [11]. Thriving is a construct that has been related to increased PYD in young people, mainly when the context provides opportunities to participate in structured, productive, and adult-led activities, such as sports or volunteering [11,16,34]. This framework is increasingly integrated with social programs and policies to promote healthy lifestyles [13,15,21].

### 1.3. The Spanish Context

Emerging adulthood is consolidating as a life stage from adolescence to 29 years old [1,2]. The Spanish Youth Institute (INJUVE) [35] showed that Spain is the country with the second-lowest rate of young people in the entire European Union. There are a total of 10,094,500 young people aged 15 to 29 (50.5% male). A total of 32% of youth lived in provincial capitals, compared to 5% who lived in towns with less than 2000 inhabitants. The number of foreign residents has increased from 3% two decades ago to 14.3%. The importance of including social policies is highlighted by the fact that 81% of young people continue to live at home, with an unemployment rate higher than that in many European countries (27% in the 15–24 age group and 17% in the 25–29 age group), with women suffering more job insecurity and temporary contracts. In addition, there is low youth participation in politics, with only almost 30% of young people showing interest. Only 47% of the sample was very interested in and committed to the environment, with women being more involved. In another study, it was also worrying that more than half of the young people did not participate in any leisure activity, despite the protective action it can have against risky behaviors such as drug use [7].

The Spanish Observatory of Drugs and Addictions [36] examined certain risk behaviors of young people aged 14–18 years throughout the country. Alcohol is the most consumed substance (73.9% said they have consumed it at least once), with an average age of onset of consumption around 14 years old. The first alcohol intoxication (binge drinking) occurs at an average age of 14.7 years, with a higher percentage in girls. Tobacco is the second most prevalent drug (38.2% have smoked at least once), with an average age of onset before 15 years old. Spanish girls showed more frequent smoking, but boys smoked a greater quantity of cigarettes. Concerning electronic cigarettes, the highest prevalence of lifetime use was found in the south of the country (around 50%). Cannabis is an illegal drug that ranks as the third most consumed drug (28.6%), has an average age of onset of 14.9 years, and is more widespread among boys. Furthermore, the prevalence in the last 12 months of betting with money was 17.2% in adolescents aged 14–18, and the prevalence of betting online was up to 9.4%. Remarkable gender differences were observed in both betting (22.7% boys, 11.6% girls) and online betting (15% boys, 3.8% girls) [36]. All these data reveal the permissive culture towards certain risky behaviors among youth [15,30].

The European Health Survey in Spain [37] also offers data for the 15–24 and 25–34 age groups about certain risk behaviors. Regarding frequent alcohol consumption (at least once a month), it is observed that it is higher in the 25–34 age group, being more frequent in men (14.7%) than in women (7.4%). Almost 10% of the population aged 15–24 years had a high consumption of alcohol at least once a month, mostly men. This frequency of consumption decreases with age, specifically among women. For men, the 25–34 age range presented the highest abuse, with approximately 14%. Lastly, regarding tobacco smoking, there has been a decrease compared to previous years, mainly in the 15–24 age group. There are more male smokers (18.3%) than female smokers (12%).

In an article by Gomez-Baya et al. [38], the authors stated that the perception of personal competence can become more important than the actual competence of different personal skills. That work concluded that girls have more emotional skills than boys, but they tend to underestimate them or demand much more from themselves than men, thus showing lower perceived emotional competence. Another study found an increased presence of externalizing problems in women, which may be due to a lower level of perception of personal competence [27]. In general, men tend to participate more in risky activities than women, and PYD may interact with other variables like parental control and family closeness [33]. Furthermore, a development marked by risky behaviors could increase the probability of suffering other concomitant problems within a cascade of risky problems across youth. Teenage pregnancy, grade repetition, and pessimistic expectations, for example, can lead these students to have higher unemployment rates, lower socioeconomic status, and drug consumption [39].

### 1.4. Purpose of the Present Study

As emerging adulthood is becoming a crucial life stage, more research on PYD beyond the age of 17 is needed to examine both risk behaviors as well as gender differences. Most of the PYD research has been conducted in the United States and other European Union countries, but more evidence with a Spanish sample is needed. Some evidence underlines that school programs based on PYD improve skills and competencies and indirectly lead to a decrease in risky behaviors [19].

The study of the PYD and its 5Cs in relation to risk behaviors in the Spanish youth population may provide some evidence to encourage program design to prevent risky behaviors, such as online gambling, drunk driving, or drug abuse. In this sense, the main aim of this work was to examine the relationship between PYD, the 5 C’s, with risk behaviors, considering gender differences. Some hypotheses were presented based on previous research. It was expected that more overall PYD (and greater scores in the 5Cs) was associated with less risky behaviors, following the line of previous research by Bonell et al. [13] and Schwartz [33]. In addition, it was also expected that males would score higher on Perceived competence and Confidence, while females would score higher on Caring and Character, consistent with the gender differences found by Manrique-Millones et al. [28] and Gomez-Baya et al. [29]. As well, more risky behaviors were expected in males, in line with data from the Spanish Observatory of Drugs and Addictions [36] and the European Health Survey in Spain [37].

## 2. Materials and Methods

### 2.1. Participants and Data Collection Procedure

The total sample consisted of 1044 young people (age range = 18–28, *M*_age_ = 20.47, *SD* = 3.08; 75.5% were female). The participants were undergraduates from 11 universities in different parts of Spain: the University of Huelva, the Loyola University (Seville and Cordoba), the Complutense University of Madrid, the University of La Laguna, the University of Salamanca, the University of Granada, the University of Zaragoza, the Polytechnic University of Valencia, the University of Santiago, the University of Valencia, and the University of Oviedo. A convenience sampling was carried out to ensure that the geographical distribution was heterogeneous and thus included universities from different regions of Spain. The choice of the degree and academic year was randomly made. The degrees were distributed into Social Sciences and Law (41.4%), Health Sciences (24.9%), Sciences and Engineering (20.2%), and Arts and Humanities (13.5%). In terms of the academic year, 27.1% of the participants were enrolled in the first year, 28.5% in the second year, 22.9% in the third year, and 21.5% in the fourth to the sixth year. Participants completed an online self-report measure. Most of the youth lived in their family home (52.8%), and only 23.2% shared an apartment with other students. Most samples stated that they were not looking for a job (63.5%). Regarding habitat, 33.9% lived in a city of more than 300,000 inhabitants, 32.2% lived in cities of 50,001–300,000 inhabitants, and the rest of the students lived in small towns or in rural areas. The exclusion criterion was reporting being 29 years old or older.

The research followed a cross-sectional design, with data collection conducted between January and May 2021. All 11 universities agreed to participate in the research. The data collection procedure was quantitative, as measured by the online survey through Qualtrics. The research team contacted and sent the necessary material to the professors and lecturers (55.1% female) of each university so that they could share it with their students. Approximately 26.4% of the contacted professors and lecturers agreed to participate in this study. If there are an average of 50 students per classroom, a total of 10,450 university students were contacted, of which 10% participated. The students answered an anonymous questionnaire with a duration of approximately 30 min within the class period, with scales related to positive youth development, lifestyles, and sociodemographic variables. Approximately 88.1% of the sample completed the survey with no omissions or less than 10 omissions. Students voluntarily agreed to participate in this study, were informed about the use of their data, and provided written informed consent. The research was approved by the Bioethics Committee of the University of Huelva on 10 January 2019. Participants did not receive any reward for participating in this study.

### 2.2. Instruments

Positive Youth Development. The PYD short form by Geldhof et al. [34] was administered. This questionnaire was piloted and adapted to Spanish by Gomez-Baya et al. [25,29] in the months of April and May 2017 with a sample of Spanish adolescents and youth. This validation study showed excellent internal consistency and reliability of the overall scale and acceptable to notable reliability in the separate subscales. The factorial validity of the scale was also observed. This instrument is composed of a total of 34 items distributed in 5 subscales in correspondence with the dimensions of the 5Cs: Perceived competence (6 items related to positive self-efficacy in different areas), Confidence (6 items related to positive self-esteem), Character (8 items, a respect for the norms of society and culture), Connection (8 items about positive relationships with others), and Caring (6 items about sympathy and empathy for others). Some examples of items respectively are: “I have a lot of friends”, “I like my physical appearance”, “I never do things I know I shouldn’t do”, and “I am a useful and important member of my family” and “It bothers me when bad things happen to other people”. Response to the items was assessed with a 5-point Likert-type scale ranging from (1) “Not at all important” to (5) “Very important”, (1) “Strongly disagree” to (5) “Strongly agree”, (1) “Not at all” to (5) “Very much”, or from (1) “Never or almost never” to (5) “Always”. An overall score resulting from the average of the above five dimensions is also calculated. Good reliability was observed in the overall scale (α = 0.88), as well as in the four Cs (Perceived competence: α = 0.73; Confidence: α = 0.77; Connection: α = 0.77; Caring: α = 0.82). The Character showed less internal consistency (α = 0.59).

Risk behaviors. A 12-item questionnaire was used, with questions related to the frequency of some risk behaviors, such as the consumption of legal drugs, the consumption of illegal drugs, the risk of accidents, and gambling. Some indicators were included for substance use, for example: “Have you consumed alcohol once or more in the last 30 days?”, “Have you been drunk once or more in the last 30 days?”, “Have you smoked a cigarette once or more in the last 30 days?”, “Have you used Vaper once or more in the last 30 days?”, “Have you smoked hookah once or more in the last 30 days?” “Have you sniffed or inhaled substances to get high once or more in the last 12 months?”, “Have you used marijuana once or more in the last 12 months?”, “Have you used other illicit drugs (e.g., cocaine, LSD, heroin, amphetamines, etc.) once or more in the last 12 months?”. Other risk behaviors, i.e., risk of accidents and betting, were also assessed: “In the last 12 months, have you driven a car or motorcycle after drinking any alcoholic beverages?”, “Have you been a passenger (once or more in the last 12 months) with a driver who has been drinking?“, “Have you bet with money once or more in the last 12 months?”, and “Have you bet online with money once or more in the last 12 months?”. Responses were coded dichotomously: Yes (1), No (0). Concerning the reliability of the scale, acceptable internal consistency was observed with KR-20 = 0.69. Exploratory factor analyses revealed a factor with an eigenvalue of 2.81, KMO = 0.71, and Bartlett’s sphericity tests were significant, χ^2^(66) = 1688.04, *p* < 0.001). Subsequently, a confirmatory factor analysis was developed and showed good data fit: Satorra-Bentler χ^2^ (47) = 60.42, *p* = 0.090, CFI = 0.984, RMSEA = 0.017, 90% CI RMSEA = 0.000, 0.029. Finally, an overall score was calculated by adding the scores in the twelve indicators of risk behaviors, which range from 0 to 12.

### 2.3. Data Analysis Design

The data were coded in SPSS 21.0, and the JASP 0.16.1.0 statistical package was used for data analysis. Listwise deletion was used to deal with missing data. First, descriptive statistics (i.e., means and standard deviations) were presented for PYD, and frequency distribution was shown for risk behaviors. Second, differences in the 5Cs and the overall PYD by each separate risk behavior (yes/no) were examined using the Student’s *t*-test. The same test was used to study gender differences. Cohen’s *d* was calculated to determine the effect size. Means and standard deviations were presented for the components of PYD based on the responses to the risk behaviors. Third, a linear regression analysis was conducted to explain the overall score in risk behaviors (ranging from 0–12), based on the scores in the 5Cs. Standardized coefficients and R^2^ were examined. Fourth, based on the previous analysis, a partial mediation model was tested to study gender differences in risk behaviors, considering gender differences in the 5Cs. Indirect, direct, and total effects were calculated. In all the statistical analyses, a significance level of *p* < 0.05 was considered.

## 3. Results

### 3.1. Descriptive Statistics of PYD and Frequencies of Risk Behaviors

Table 1 shows the descriptive statistics (mean and standard deviation) of PYD, as well as the risk behavior’s frequencies (percentage of participants answering “Yes”), in the total sample and by gender. The results indicated a moderate overall PYD (*M* = 3.71; *SD* = 0.42) on a range of scores from 1 to 5. The highest values within the 5Cs of PYD were observed for Caring and Character, while the lowest score was detected in Perceived competence. Women reported more Caring and Character, but less perceived Competence than men.

Regarding risk behaviors, more than 70% of the sample reported alcohol consumption in the last 30 days, with no gender differences. Around one-third of the sample reported alcohol drunkenness, with greater frequency among men. Approximately 11.7% of the participants had driven a car or motorcycle under the influence of alcohol (more frequent among men), and 27.2% had been occupants of a vehicle whose driver was drunk. Moreover, nearly 30% of the sample had smoked in the last 30 days, while around 3% indicated having used an electronic cigarette (vaper). Hookah smoking was used by 6.4% of the sample, with no gender differences. With regards to other drugs, 2.7% said they had used them in the last year, while 20% had used cannabis and 3.3% had sniffed substances. More frequent consumption of other drugs was observed in men. Finally, in relation to gambling, around 6% of the sample had gambled with money in the last year, while around 4% had gambled online, both behaviors being more practiced in the sample of men.

### 3.2. Differences in PYD by the Responses in the Indicators of Risk Behaviors

Table 2 shows mean differences in the overall PYD and its 5Cs by the response in the indicators of alcohol use, drunkenness, and tobacco use, as well as their differences by gender. First, participants who have not drunk alcohol in the last 30 days showed higher Character scores. However, those who had drunk some alcohol showed more Perceived competence and Connection. Considering gender, women who have drunk some alcohol scored higher on Perceived competence, while women who did not drink some alcohol scored more on Character. Men who did drink some alcohol had more Perceived competence and Connection. According to alcohol drunkenness in the total sample, those who had gotten drunk in the last 30 days showed more Perceived competence but lower Character. The same pattern was found for women on Perceived competence and Character. Men who got drunk also presented higher Perceived competence. On tobacco use in the last 30 days, those participants who had not smoked scored higher on Character, while those who smoked presented higher Perceived competence. Considering gender, girls who did not smoke showed higher scores on Character and Confidence, whereas men who did smoke scored more on Perceived competence. No differences were found in the overall PYD score for any of the risky behaviors mentioned. All effect size values were small for the statistically significant results.

Table 3 shows the Student *t*-test results regarding vaper use (i.e., electronic cigarettes), smoking hookah, and sniffing substances. It was found that those participants who did not inhale any substance in the last 12 months showed better Character, presenting a small effect size. By gender, it was found that women who had not conducted it indicated greater Confidence, with a medium effect size. No differences were found in the overall PYD score in substance sniffing, as well as no statistically significant differences in any C of PYD for smoking hookah and vaper use. 

The results of the Student *t*-test of overall PYD and its components by cannabis abuse, the use of other drugs, and drunk driving are described in Table 4. First, the results indicated that youth who had not used cannabis in the past 12 months scored higher on Character and Connection, whereas Perceived competence was higher for those who had smoked cannabis. Examining the results by gender, women who used cannabis reported higher Perceived competence, while both men and women who had not used cannabis scored higher on Character. Second, regarding illegal drug use in the whole sample, those who used them showed higher Perceived competence. These results were only found in men. Third, drunk driving was associated with greater Perceived competence and Confidence, and less Character. Both men and women reported greater Perceived competence if they reported drunk driving. Only women who did not drive drunk had more Character, while men who drove drunk had higher Confidence scores. No differences were found in the overall PYD score for any of the variables. All effect size values were small, except for Perceived competence in men with drug use and drunk driving, where a medium effect size was observed.

Finally, Table 5 presents the results of the indicators of being a passenger in a car driven by someone who has been drunk and gambling with money online. Those who had been passengers of someone who drove drunk in the last 12 months reported more Perceived competence, with a small effect size. The same results were observed in women, with a small effect size, and in men, with a medium effect size. In addition, there was greater overall PYD in men who were passengers of a drunk driver, with a small effect size.

Furthermore, those participants who bet money and made online bets in the last year scored higher on Perceived competence, both with a medium effect size. Furthermore, those who did not bet with either money (with a medium effect size) or online betting (with a small effect size) scored higher on Caring. Moreover, those who stayed away from money gambling scored higher on Character (with a small effect size) but less connection. Online betting was inversely related to confidence in the overall sample. By gender, women and men who bet money reported greater Perceived competence (medium effect size). In addition, men reported greater Connection (small effect size), while those men who did not gamble had more Caring (medium effect size). Concerning online betting, those men who reported having conducted so scored higher in Perceived competence, Confidence, and Connection. However, more Caring was found in men who did not gamble online. No significant results were observed in women with PYD concerning online gambling. No statistically significant differences in overall PYD were found in relation to gambling with money or online gambling.

### 3.3. Linear Regression Analysis to Explain Overall Score in Risk Behaviors Based on the 5Cs

Table 6 presents the results of the linear regression analysis to explain the overall score in the risk behaviors (*M* = 2.21, *SD* = 1.92). This overall score was calculated by adding the twelve separated indicators, which were coded 1 (yes) and 0 (no), so that a higher score indicates a greater presence of risky behaviors. The model reached above ten percent of the variance of the overall score, based on gender, age, and the 5Cs. Gender differences were detected, *t* (964) = 4.49, *p* < 0.001, *d* = 0.34, so that men (*M* = 2.69, *SD* = 2.31) presented higher scores than women (*M* = 2.05, *SD* = 1.74). No age differences were observed, and no significant effects were found in confidence, caring, or connection. Furthermore, a positive effect of perceived competence and a negative one of character were detected on the overall score of risk behaviors. Thus, more perceived competence and less character were associated with more risky behaviors.

### 3.4. Partial Mediation Model to Explain Gender Differences in Risk Behaviors Based on Gender Differences in the Significant Cs

**Figure 1:**
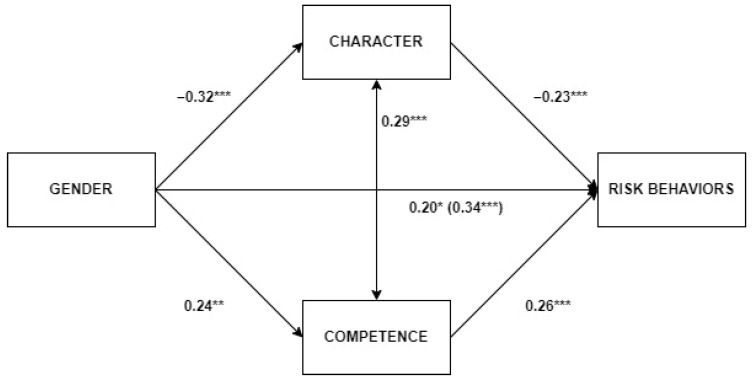
Partial mediation model with standardized coefficients. Note: *** *p* < 0.001; ** *p* < 0.01; * *p* < 0.05.

Table 7 shows the results of the partial mediation analysis of the relationship between gender and the overall score in risk behaviors through the effects on character and perceived competence. Figure 1 represents the standardized coefficients of the relationships included in the model. The results indicated that the effect of gender on risk behaviors was partly mediated by the indirect effects of character and perceived competence. The gender effect on risk behaviors remained significant after the inclusion of both mediators (i.e., character and perceived competence). The total effect of gender on risk behaviors (before including the mediators) was β = 0.34, *p* < 0.001, and it was reduced to β = 0.20, *p* = 0.006. Thus, gender differences in risk behaviors (with females presenting lower scores) were partly due to their greater scores in character and their lower scores in perceived competence. As well, a positive interrelation was observed between character and perceived competence.

## 4. Discussion

The aim of this study was to examine the associations between the overall PYD and its 5Cs regarding several risk behaviors, such as substance use (alcohol, tobacco, other drugs, and others) and gambling, as well as gender differences in those associations. In general, when examining the overall score in risk behaviors, the results indicated that the dimensions of character and perceived competence had the strongest effects. Thus, the presence of risky behaviors was associated with less character and more perceived competence. If we examined the separate risk behaviors, some interesting results were also observed. Following our initial hypothesis, we found that a higher score in certain components of the 5Cs was associated with a transition to adulthood marked by a lifestyle away from risky behaviors. A lifestyle away from alcohol, drunkenness, smoking, gambling, and other drugs, as well as drunk driving or being a passenger of a drunk driver, was associated with better Character, mainly in women. This component of the 5Cs showed high relevance, most likely due to an appropriate internalization of behavioral norms according to social norms and culture [17,23]. More Caring was also presented to those who did not participate in gambling with money or online in the last year. These findings about the protective role of PYD are in line with the research by Gomez-Baya et al. [25,29] and the works by Bonell et al. [13] and Schwartz [33]. These components, mainly Character, have proven to be a solid foundation for moving away from certain risky behaviors to achieve optimal development in young people.

However, some contradictory and unexpected results were found concerning Perceived competence. It was expected that the Perceived competence component would be higher in those who did not participate in any risky behavior, but that was not the case. Higher levels of Perceived competence were associated with the practice of some risky behaviors included in this study, which may contradict the findings of several studies that concluded that the higher the number of PYD components, the lower the number of problem behaviors [26,27,29,32]. There are several possible explanations for this, based on previous literature. First, in line with some authors [32,39], some possible factors that may alter healthy development could not have been considered in this study, such as the presence of pessimistic expectations, the socioeconomic level available, a low level of education, a low level of social support, or other psychosocial factors that may influence PYD. Second, in Western countries, the consumption of certain substances such as alcohol and tobacco has been associated with celebration, parties, and enjoyment, becoming socially acceptable, and what may become drugs in a way to promote the social component of Perceived competence and Connection. In turn, those Cs were also related to the participation of betting money and online betting among men in this study [33]. Third, this consumption may not be considered problematic by some youth, and even they may consider it an opportunity to discover who they are and what they want. Fourth, it is possible that, following Schwartz [33], a combination of different Cs is needed to be able to have a protective profile and that only some of these components are significantly related to some specific risk behaviors. Fifth, uneven development of the components may affect the lifestyles of young people, since the exaggerated positive view of themselves, coupled with low Confidence and Character, could lead to a biased awareness of danger when engaging in certain behaviors. Finally, another possible explanation could be the lack of leisure and free time activities available to the young people in our sample, since this lack of opportunities for healthier leisure time would be a risk factor for engaging in these types of behaviors [11,16,19].

Regarding the gender differences in relation to the 5Cs, it was hypothesized that the male sample would score higher on Perceived competence and Confidence, while the female sample would score higher on Caring and Character. As well, gender differences were observed in risk behaviors, so men reported more risk behaviors than women. This result was consistent with the hypothesis, based on Manrique-Millones et al. [28] and Gomez-Baya et al. [29]. The results of a partial mediation model in the present study showed that these gender differences in risk behaviors were partly due to gender differences in character and perceived competence. Thus, higher scores in perceived competence and lower character partly explained the presence of more risky behaviors in males. Due to gender stereotypes present in society, men tend to be more focused on achievement, while women are taught to be more emotionally and socially oriented [7,28]. Similarly, women tend to underestimate their abilities, and men overestimate them [38]. In the same vein, Schwartz et al. [33] argued that men participated more in risky activities. However, following Bonell et al. [13], more research is needed to understand the relationships between PYD and healthy lifestyles, which would be formed by components of health protection and the absence of risky behaviors. In this line, another study [32] highlights that the benefits of interventions based on PYD models are not clear, specifically those focused on sexual health. However, other studies affirm that a greater presence of PYD components, as well as a higher overall PYD, leads to greater personal well-being and that of others [15,25,26]. Likewise, it would be of great interest for future studies to consider self-efficacy beliefs, which have been positively associated with PYD since they encourage youth to become active agents in the face of adversity and have been related to the assumption of lifestyles without risky behaviors [15].

### 4.1. Limitations

There are limitations to this work that should be pointed out for future research in this area. First, the Spanish universities were chosen by convenience, and access to the sample was mediated by the faculty staff of each institution, resulting in less than half of the professors and lecturers contacted agreeing to participate in this research. Moreover, the low participation of the students of the professors/lecturers who shared the questionnaire may produce some bias in the results. Secondly, there was a higher representation of female participants (75.5%), which indicates that future research should be more gender-balanced. Additionally, self-report measures were used, so only subjective information was collected. Thirdly, although significant differences were found, it is important to emphasize that most of these differences had a small effect size. In fourth place, although anonymity gives the opportunity to respond without judgment, some social desirability may bias the responses of young people, resulting in a lack of objectivity or unwillingness to affirm that they engage in behaviors against social norms. Another limitation is related to the cross-sectional design of this study, since conclusions only establish associations between the study variables at a specific time. For this study of emerging adulthood, the use of longitudinal designs is advisable to examine how the PYD can predict certain risk behaviors as well as the emergence of possible gender differences. Furthermore, for future studies, it would be interesting to add Contribution, the 6th C of the PYD, which refers to the feeling of involvement beyond oneself, providing support to the family, community, or environment. In this line, other variables should be jointly examined, following an ecological model, including variables such as genetic factors, family characteristics, and national power. Moreover, future research should analyze the mechanisms between PYD and risk behaviors, including critical thinking, coping styles, or social skills, as competences to deal successfully with risks. A better understanding of the associations between PYD and risk behaviors will make intervention designs more accurate.

### 4.2. Implications for Research, Policy, and Practice

Even with the above limitations in mind, this study has significant implications for research, policy, and practice. One implication for research is related to the importance of treating PYD not only as an overall factor but in a multidimensional way to observe the impact of the 5Cs on young undergraduates. Further analysis is needed to link each component to a specific risk behavior since it seems that elevated levels of Perceived competence can become maladaptive despite being an adaptive component, according to the scientific literature [15,25]. Second, public policies should address the vulnerability that many young people may suffer in terms of little or no participation in healthy leisure time activities in different contexts (family, educational, and community). Other research has shown that leisure programs based on PYD favor healthy development while reducing risky behaviors [7]. In addition, universities should include in intervention programs good practices for healthy lifestyles, such as recommendations and advice. A good example of this can be found in the Spanish Network of Health-Promoting Universities [40]. Finally, an implication for practice is to use the 5Cs indicators (i.e., Character) to create intervention strategies to promote healthy and optimal development, far away from risky behaviors. Therefore, the present study underlines that both professionals and researchers should work together to promote PYD, considering the separate effects of the 5Cs. The results of this study highlight the need to design gender-based interventions to jointly promote PYD and healthy lifestyles by nurturing character and adaptive self-efficacy beliefs.

## 5. Conclusions

Youth is an important transitional life stage that determines the foundations for adult life. PYD is a strengths-based model that encourages the development of opportunities and resources to promote healthy lifestyles away from risky behaviors. Most of the research has been conducted with North American samples, which shows the relevance of conducting similar studies in other contexts, such as Spain. Such studies may be useful to establish intervention programs that favor healthy development.

This research adds some evidence to the literature on PYD, exploring knowledge about the 5Cs and overall PYD in relation to certain risk behaviors. First, it was found that Character was positively associated with the absence of risky behaviors, a finding that may indicate where prevention programs should pay closer attention. Second, Perceived competence was found to be positively associated with risk behaviors, which may indicate that very high levels of Perceived competence can become a risk factor that should be considered in addressing this problem in young undergraduates. Third, it was observed that gender differences in the presence of risk behaviors were partly due to gender differences in Character and Perceived competence. Consequently, the results obtained in this research can help in research, policies, and programs that seek to improve the healthy development of Spanish youth while protecting gender equality. Further research is needed to replicate these results and determine their protective and predictive value in the short and long term. More studies are needed that include self-efficacy beliefs and objective measures of competence. If risky behaviors are not addressed in time, they could have serious personal and community consequences. Thus, it is important to design and implement PYD programs to promote healthy lifestyles by addressing gender differences and nurturing Character and adaptive Perceived competence.

## Figures and Tables

**Table 1:** PYD descriptive statistics and frequency distribution of risk behaviors in the total sample and by gender.

PYD	*M* (*SD*)	*M* (*SD*) Women	*M* (*SD*) Men	*t*-Test
Character	4.00 (0.43)	4.04 (0.41)	3.90 (0.46)	4.40 ***
Perceived competence	3.01 (0.71)	2.96 (0.69)	3.14 (0.78)	−3.30 **
Confidence	3.64 (0.69)	3.62 (0.70)	3.71 (0.68)	−1.71
Caring	4.32 (0.59)	4.39 (0.55)	4.08 (0.64)	7.38 ***
Connection	3.61 (0.61)	3.63 (0.60)	3.56 (0.65)	1.57
Overall PYD	3.71 (0.42)	3.73 (0.41)	3.67 (0.45)	1.70
Risk behaviors	% Yes overall	% Yes women	% Yes men	*Χ* ^2^
Alcohol use	71.5	71.1	72.5	0.16
Drunkenness	34.6	31.2	45.4	16.34 ***
Smoking	28.6	29.0	27.4	0.23
Vaper use	3.3	3.1	4.1	0.66
Smoking hookah	6.8	6.8	6.6	0.01
Sniffing substances	3.3	2.4	6.6	10.77 **
Cannabis use	20.0	17.2	28.8	15.01 ***
Other drugs use	2.7	1.5	6.7	18.49 ***
Drunk driving	11.7	10.3	16.3	6.33 *
Passenger with a drunk driver	27.2	27.1	27.4	0.01
Bet money	6.1	2.9	15.8	50.72 ***
Online betting	3.9	1.9	10.4	35.26 ***

Note: *** *p* < 0.001; ** *p* < 0.01; * *p* < 0.05.

**Table 2:** Results of *t*-tests based on the mean scores for PYD by the responses to the indicators of alcohol use, drunkenness, and smoking.

			Alcohol Use			Drunkenness				Smoking			
		Yes	No			Yes	No			Yes	No		
		*M* (*SD*)	*M* (*SD*)	*t*	*d*	*M* (*SD*)	*M* (*SD*)	*t*	*d*	*M* (*SD*)	*M* (*SD*)	*t*	*d*
Overall	Character	3.97 (0.44)	4.07 (0.44)	3.49 ***	0.25	3.91 (0.42)	4.05 (0.42)	4.96 ***	0.33	3.92 (0.41)	4.03 (0.43)	3.57 ***	0.25
	Perceived competence	3.07 (0.68)	2.84 (0.74)	−4.55 ***	−0.32	3.14 (0.68)	2.93 (0.71)	−4.59 ***	−0.31	3.10 (0.69)	2.96 (0.71)	−2.62 **	−0.18
	Confidence	3.64 (0.67)	3.64 (0.75)	−0.03	−0.00	3.64 (0.63)	3.63 (0.71)	−0.16	−0.01	3.58 (0.70)	3.66 (0.69)	1.72	0.12
	Caring	4.32 (0.55)	4.30 (0.66)	−0.32	−0.02	4.28 (0.59)	4.33 (0.58)	1.32	0.09	4.36 (0.56)	4.29 (0.59)	−1.69	−0.12
	Connection	3.64 (0.59)	3.55 (0.64)	−2.03 *	−0.14	3.61 (0.58)	3.61 (0.63)	0.13	0.01	3.62 (0.56)	3.61 (0.63)	−0.33	−0.02
	Overall	3.72 (0.40)	3.68 (0.46)	−1.43	−0.10	3.72 (0.40)	3.71 (0.43)	−0.18	−0.01	3.71 (0.40)	3.71 (0.42)	−0.11	−0.01
Women	Character	4.00 (0.40)	4.11 (0.42)	3.30 **	0.27	3.93 (0.41)	4.08 (0.40)	4.39 ***	0.35	3.94 (0.41)	4.07 (0.41)	3.84 ***	0.31
	Perceived competence	3.02 (0.66)	2.83 (0.71)	−3.48 ***	−0.28	3.08 (0.65)	2.91 (0.69)	−3.31 ***	−0.26	3.03 (0.66)	2.94 (0.69)	−1.66	−0.13
	Confidence	3.61 (0.68)	3.64 (0.74)	0.60	0.05	3.59 (0.65)	3.63 (0.72)	0.60	0.05	3.54 (0.72)	3.65 (0.69)	1.98 *	0.16
	Caring	4.39 (0.53)	4.38 (0.59)	−0.17	−0.01	4.39 (0.56)	4.39 (0.54)	−0.06	−0.00	4.43 (0.54)	4.37 (0.55)	−1.24	−0.10
	Connection	3.64 (0.59)	3.59 (0.61)	−1.04	−0.08	3.62 (0.55)	3.63 (0.62)	0.36	0.03	3.60 (0.54)	3.64 (0.61)	0.70	0.06
	Overall	3.73 (0.40)	3.71 (0.44)	−0.58	−0.05	3.72 (0.39)	3.73 (0.42)	0.07	0.01	3.71 (0.41)	3.73 (0.41)	0.81	0.07
Men	Character	3.87 (0.43)	3.95 (0.49)	1.26	0.18	3.85 (0.44)	3.93 (0.46)	1.44	0.19	3.86 (0.42)	3.91 (0.46)	0.75	0.11
	Perceived competence	3.22 (0.73)	2.89 (0.83)	−2.98 **	−0.43	3.27 (0.77)	3.01 (0.77)	−2.63 **	−0.34	3.32 (0.74)	3.05 (0.78)	−2.40 *	−0.35
	Confidence	3.73 (0.65)	3.62 (0.76)	−1.13	−0.16	3.75 (0.66)	3.66 (0.69)	−0.93	−0.12	3.71 (0.65)	3.70 (0.69)	−0.08	−0.01
	Caring	4.09 (0.55)	4.04 (0.81)	−0.51	−0.07	4.04 (0.57)	4.11 (0.68)	0.83	0.11	4.15 (0.57)	4.05 (0.65)	−1.12	−0.16
	Connection	3.62 (0.62)	3.40 (0.70)	−2.26 *	−0.33	3.59 (0.63)	3.53 (0.66)	−0.65	−0.09	3.67 (0.60)	3.51 (0.66)	−1.76	−0.25
	Overall	3.71 (0.41)	3.59 (0.51)	−1.87	−0.27	3.70 (0.42)	3.65 (0.46)	−0.82	−0.11	3.74 (0.39)	3.64 (0.46)	−1.51	−0.22

Note: *** *p* < 0.001; ** *p* < 0.01; * *p* < 0.05.

**Table 3:** Results of *t*-tests based on the mean scores for PYD by the responses to the indicators of vaper use, smoking hookah, and sniffing substances.

			Vaper Use			Smoking Hookah				Sniffing Substances			
		Yes	No			Yes	No			Yes	No		
		*M* (*SD*)	*M* (*SD*)	*t*	*d*	*M* (SD)	*M* (*SD*)	*t*	*d*	*M* (*SD*)	*M* (*SD*)	*t*	*d*
Overall	Character	4.07 (0.40)	3.99 (0.42)	−1.05	−0.19	3.96 (0.42)	4.00 (0.42)	0.72	0.09	3.83 (0.42)	4.00 (0.42)	2.29 *	0.41
	Perceived competence	3.14 (0.66)	3.00 (0.71)	−1.10	−0.20	2.99 (0.67)	3.00 (0.71)	0.16	0.02	3.15 (0.77)	3.00 (0.71)	−1.19	−0.21
	Confidence	3.72 (0.62)	3.63 (0.70)	−0.69	−0.12	3.68 (0.58)	3.63 (0.70)	−0.54	−0.07	3.46 (0.74)	3.64 (0.69)	1.53	0.27
	Caring	4.30 (0.48)	4.31 (0.59)	0.14	0.02	4.32 (0.69)	4.31 (0.58)	−0.05	−0.01	4.22 (0.59)	4.32 (0.59)	0.89	0.16
	Connection	3.81 (0.66)	3.60 (0.61)	−1.90	−0.34	3.65 (0.65)	3.61 (0.61)	−0.54	−0.07	3.44 (0.58)	3.62 (0.61)	1.58	0.28
	Overall	3.81 (0.38)	3.71 (0.42)	−1.32	−0.23	3.72 (0.43)	3.71 (0.42)	−0.15	−0.02	3.62 (0.40)	3.72 (0.42)	1.27	0.23
Women	Character	4.04 (0.39)	4.03 (0.41)	−0.13	−0.03	3.97 (0.37)	4.04 (0.41)	1.01	0.15	3.86 (0.44)	4.04 (0.41)	1.76	0.43
	Perceived competence	3.10 (0.52)	2.96 (0.69)	−0.95	−0.20	2.96 (0.59)	2.96 (0.69)	−0.01	−0.00	2.96 (0.85)	2.96 (0.68)	0.03	0.01
	Confidence	3.67 (0.54)	3.61 (0.70)	−0.38	−0.08	3.64 (0.57)	3.61 (0.71)	−0.25	−0.04	3.27 (0.83)	3.63 (0.69)	2.07 *	0.51
	Caring	4.37 (0.49)	4.39 (0.55)	0.14	0.03	4.46 (0.56)	4.38 (0.55)	−0.87	−0.13	4.49 (0.45)	4.39 (0.55)	−0.73	−0.18
	Connection	3.79 (0.56)	3.62 (0.60)	−1.31	−0.28	3.73 (0.57)	3.62 (0.60)	−1.22	−0.18	3.48 (0.65)	3.63 (0.59)	1.07	0.26
	Overall	3.79 (0.31)	3.72 (0.41)	−0.81	−0.17	3.75 (0.35)	3.72 (0.41)	−0.48	−0.07	3.61 (0.50)	3.73 (0.41)	1.17	0.29
Men	Character	4.15 (0.44)	3.88 (0.45)	−1.83	−0.59	3.92 (0.56)	3.89 (0.44)	−0.26	−0.07	3.80 (0.42)	3.90 (0.45)	0.82	0.21
	Perceived competence	3.23 (0.94)	3.12 (0.77)	−0.43	−0.14	3.07 (0.92)	3.13 (0.77)	0.30	0.08	3.35 (0.64)	3.11 (0.78)	−1.19	−0.31
	Confidence	3.83 (0.81)	3.69 (0.67)	−0.62	−0.20	3.81 (0.62)	3.69 (0.68)	−0.66	−0.17	3.65 (0.58)	3.70 (0.69)	0.29	0.07
	Caring	4.13 (0.42)	4.07 (0.65)	−0.27	−0.09	3.88 (0.89)	4.09 (0.61)	1.27	0.33	3.94 (0.61)	1.08 (0.63)	0.86	0.22
	Connection	3.86 (0.86)	3.54 (0.64)	−1.49	−0.48	3.40 (0.81)	3.57 (0.64)	0.95	0.25	3.41 (0.52)	3.56 (0.66)	0.90	0.23
	Overall	3.84 (0.52)	3.66 (0.44)	−1.21	−0.39	3.62 (0.61)	3.67 (0.43)	0.50	0.13	3.63 (0.26)	3.67 (0.45)	0.36	0.09

Note: * *p* < 0.05.

**Table 4:** Results of *t*-tests based on the mean scores for PYD by the responses to the indicators of cannabis use, drug use, and drunk driving.

			Cannabis Use			Drugs Use				Drunk Driving			
		Yes	No			Yes	No			Yes	No		
		*M* (*SD*)	*M* (*SD*)	*t*	*d*	*M* (*SD*)	*M* (*SD*)	*t*	*d*	*M* (*SD*)	*M* (*SD*)	*t*	*d*
Overall	Character	3.90 (0.43)	4.02 (0.42)	3.76 ***	0.30	3.88 (0.40)	4.00 (0.42)	1.43	0.28	3.92 (0.40)	4.01 (0.43)	2.18 *	0.28
	Perceived competence	3.13 (0.68)	2.97 (0.71)	−2.91 **	−0.23	3.30 (0.75)	2.99 (0.71)	−2.23 *	−0.44	3.29 (0.65)	2.96 (0.71)	−4.68 ***	−0.46
	Confidence	3.58 (0.71)	3.65 (0.69)	1.33	0.11	3.64 (0.73)	3.64 (0.69)	−0.06	−0.01	3.77 (0.59)	3.62 (0.71)	−2.22 *	−0.22
	Caring	4.27 (0.63)	4.33 (0.57)	1.18	0.09	4.28 (0.56)	4.31 (0.59)	0.26	0.05	4.23 (0.62)	4.32 (0.58)	1.54	0.15
	Connection	3.53 (0.62)	3.63 (0.60)	2.01 *	0.16	3.51 (0.64)	3.61 (0.61)	0.84	0.17	3.66 (0.50)	3.60 (0.62)	−0.98	−0.10
	Overall	3.68 (0.41)	3.72 (0.42)	1.23	0.10	3.71 (0.38)	3.71 (0.04)	0.05	0.01	3.77 (0.37)	3.70 (0.43)	−1.70	−0.17
Women	Character	3.95 (0.43)	4.05 (0.41)	2.55 *	0.25	3.99 (0.36)	4.03 (0.41)	0.36	0.11	3.94 (0.39)	4.04 (0.41)	1.97 *	0.24
	Perceived competence	3.09 (0.63)	2.93 (0.69)	−2.35 *	−0.23	3.01 (0.86)	2.96 (0.68)	−0.23	−0.07	3.15 (0.66)	2.94 (0.68)	−2.59 *	−0.31
	Confidence	3.53 (0.74)	3.63 (0.69)	1.45	0.14	3.56 (0.83)	3.62 (0.70)	0.28	0.08	3.71 (0.62)	3.61 (0.71)	−1.21	−0.15
	Caring	4.40 (0.56)	4.39 (0.55)	−0.21	−0.02	4.58 (0.52)	4.39 (0.55)	−1.09	−0.35	4.40 (0.54)	4.39 (0.55)	−0.26	−0.03
	Connection	3.55 (0.61)	3.65 (0.59)	1.73	0.17	3.41 (0.69)	3.63 (0.59)	1.17	0.37	3.70 (0.48)	3.62 (0.61)	−1.15	−0.14
	Overall	3.70 (0.42)	3.73 (0.41)	0.78	0.08	3.66 (0.42)	3.73 (0.41)	0.50	0.16	3.78 (0.41)	3.72 (0.41)	−1.27	−0.15
Men	Character	3.80 (0.44)	3.93 (0.45)	1.98 *	0.28	3.81 (0.42)	3.90 (0.45)	0.76	0.20	3.86 (0.41)	3.90 (0.46)	0.43	0.08
	Perceived competence	3.21 (0.76)	3.09 (0.45)	−1.07	−0.15	3.51 (0.62)	3.10 (0.78)	−2.02 *	−0.52	3.56 (0.53)	3.04 (0.79)	−3.90 ***	−0.68
	Confidence	3.66 (0.64)	3.72 (0.69)	0.63	0.09	3.70 (0.67)	3.70 (0.68)	−0.01	−0.00	3.90 (0.51)	3.66 (0.70)	−2.00 *	−0.35
	Caring	4.03 (0.68)	4.09 (0.62)	0.71	0.10	4.10 (0.52)	4.07 (0.64)	−0.15	−0.04	3.90 (0.63)	4.11 (0.63)	1.92	0.34
	Connection	3.51 (0.66)	3.57 (0.65)	0.70	0.10	3.58 (0.62)	3.56 (0.65)	−0.12	−0.03	3.58 (0.54)	3.55 (0.67)	−0.32	−0.06
	Overall	3.64 (0.40)	3.68 (0.46)	0.66	0.09	3.74 (0.36)	3.67 (0.45)	−0.61	−0.16	3.76 (0.27)	3.65 (0.47)	−1.38	−0.24

Note: *** *p* < 0.001; ** *p* < 0.01; * *p* < 0.05.

**Table 5:** Results of *t*-tests based on the mean scores for PYD by the responses on being a passenger with a drunk driver, betting money, and online betting.

		Passenger with a Drunk Driver				Bet Money				Online Betting			
		Yes	No			Yes	No			Yes	No		
		*M* (*SD*)	*M* (*SD*)	*t*	*d*	*M* (*SD*)	*M* (*SD*)	*t*	*d*	*M* (*SD*)	*M* (*SD*)	*t*	*d*
Overall	Character	3.96 (0.43)	4.01 (0.42)	1.86	0.13	3.84 (0.45)	4.01 (0.42)	2.84 **	0.38	3.95 (0.43)	4.00 (0.42)	0.66	0.11
	Perceived competence	3.15 (0.69)	2.95 (0.71)	−4.13 ***	−0.30	3.45 (0.70)	2.97 (0.70)	−5.06 ***	−0.68	3.37 (0.60)	2.99 (0.71)	−3.24 **	−0.54
	Confidence	3.63 (0.64)	3.64 (0.71)	0.25	0.02	3.79 (0.68)	3.63 (0.69)	−1.76	−0.24	3.88 (0.55)	3.63 (0.70)	−2.18 *	−0.36
	Caring	4.31 (0.58)	4.32 (0.59)	0.20	0.01	3.99 (0.79)	4.33 (0.56)	4.37 ***	0.59	4.07 (0.70)	4.32 (0.58)	2.64 **	0.44
	Connection	3.64 (0.54)	3.60 (0.63)	−1.08	−0.08	3.78 (0.65)	3.60 (0.61)	−2.16*	−0.29	3.75 (0.71)	3.60 (0.60)	−1.44	−0.24
	Overall	3.74 (0.39)	3.70 (0.43)	−1.10	−0.08	3.77 (0.48)	3.71 (0.42)	−1.10	−0.15	3.80 (0.35)	3.71 (0.42)	−1.34	−0.22
Women	Character	3.98 (0.43)	4.05 (0.40)	1.92	0.16	3.90 (0.45)	4.04 (0.41)	1.54	0.33	4.05 (0.45)	4.03 (0.41)	−0.15	−0.04
	Perceived competence	3.05 (0.67)	2.93 (0.68)	−2.25 *	−0.19	3.33 (0.77)	2.95 (0.68)	−2.56 *	−0.55	3.11 (0.46)	2.96 (0.68)	−0.79	−0.22
	Confidence	3.56 (0.65)	3.64 (0.72)	1.31	0.11	3.61 (0.82)	3.62 (0.70)	0.05	0.01	3.59 (0.72)	3.62 (0.70)	0.16	0.04
	Caring	4.40 (0.53)	4.39 (0.55)	−0.27	−0.02	4.43 (0.60)	4.39 (0.55)	−0.40	−0.09	4.56 (0.52)	4.39 (0.55)	−1.13	−0.32
	Connection	3.64 (0.54)	3.62 (0.62)	−0.40	−0.03	3.77 (0.63)	3.62 (0.59)	−1.14	−0.25	3.61 (0.88)	3.63 (0.59)	0.10	0.03
	Overall	3.73 (0.40)	3.72 (0.41)	−0.02	−0.00	3.81 (0.51)	3.72 (0.41)	−0.94	−0.20	3.78 (0.42)	3.72 (0.41)	−0.50	−0.14
Men	Character	3.87 (0.42)	3.90 (0.46)	0.44	0.06	3.81 (0.45)	3.91 (0.45)	1.16	0.21	3.90 (0.43)	3.89 (0.45)	−0.12	−0.02
	Perceived competence	3.46 (0.67)	3.00 (0.78)	−4.20 ***	−0.61	3.52 (0.66)	3.05 (0.78)	−3.45 ***	−0.62	3.50 (0.63)	3.08 (0.78)	−2.56 *	−0.54
	Confidence	3.83 (0.59)	3.65 (0.71)	−1.85	0.27	3.90 (0.58)	3.66 (0.69)	−1.96	−0.35	4.03 (0.38)	3.66 (0.69)	−2.61 *	−0.55
	Caring	4.03 (0.65)	4.09 (0.63)	0.70	0.10	3.73 (0.78)	4.14 (0.58)	3.73 ***	0.67	3.81 (0.65)	4.10 (0.62)	2.21 *	0.47
	Connection	3.65 (0.56)	3.52 (0.68)	−1.42	−0.21	3.78 (0.66)	3.51 (0.64)	−2.31 *	−0.41	3.82 (0.61)	3.52 (0.65)	−2.17 *	−0.46
	Overall	3.77 (0.37)	3.63 (0.46)	−2.10 *	−0.30	3.75 (0.46)	3.66 (0.44)	−1.16	−0.21	3.81 (0.31)	3.65 (0.46)	−1.69	−0.36

Note: *** *p* < 0.001; ** *p* < 0.01; * *p* < 0.05.

**Table 6:** Results of linear regression analysis.

	Durbin-Watson	R^2^	F	*t*	β
Model	1.93	0.11	18.13 ***		
Gender				3.25	0.10 **
Age				−0.87	−0.03
Character				6.33	−0.25 ***
Perceived Competence				7.49	0.30 ***
Confidence				−1.68	−0.07
Caring				1.95	0.07
Connection				0.09	0.01

Note: *** *p* < 0.001; ** *p* < 0.01.

**Table 7:** Results of the partial mediation model.

					95% Confidence Interval	
	Estimate	Std. Error	Z Value	*p*	Lower	Upper
Direct effect						
Gender → Risk behavior	0.20	0.07	2.73	0.006	0.06	0.34
Indirect effects						
Gender → Character → Risk behavior	0.08	0.02	3.76	<0.001	0.04	0.11
Gender → Perceived competence → Risk behavior	0.06	0.02	3.06	0.002	0.02	0.10
Total effect						
Gender → Risk behavior	0.34	0.07	4.53	<0.001	0.19	0.48
Total indirect effect						
Gender → Risk behavior	0.14	0.03	5.29	<0.001	0.09	0.19
Residual covariance						
Character ↔ Perceived competence	0.29	0.03	8.86	<0.001	0.22	0.35

Note: Risk behaviors: R^2^ = 0.11; Character: R^2^ = 0.02; Perceived competence: R^2^ = 0.01.

## Data Availability

The raw data supporting the conclusions of this article will be made available by the authors without undue reservation.
